# Transformer-based deep neural network language models for Alzheimer’s disease risk assessment from targeted speech

**DOI:** 10.1186/s12911-021-01456-3

**Published:** 2021-03-09

**Authors:** Alireza Roshanzamir, Hamid Aghajan, Mahdieh Soleymani Baghshah

**Affiliations:** 1 Department of Computer Engineering, Sharif University of Technology, Azadi Avenue, Tehran, Iran; 2 Department of Electrical Engineering, Sharif University of Technology, Azadi Avenue, Tehran, Iran

**Keywords:** Alzheimer’s disease, Early risk assessment, Picture description test, Deep learning, Transformer, Natural language processing, Language model, Transfer learning

## Abstract

**Background:**

We developed transformer-based deep learning models based on natural language processing for early risk assessment of Alzheimer’s disease from the picture description test.

**Methods:**

The lack of large datasets poses the most important limitation for using complex models that do not require feature engineering. Transformer-based pre-trained deep language models have recently made a large leap in NLP research and application. These models are pre-trained on available large datasets to understand natural language texts appropriately, and are shown to subsequently perform well on classification tasks with small training sets. The overall classification model is a simple classifier on top of the pre-trained deep language model.

**Results:**

The models are evaluated on picture description test transcripts of the Pitt corpus, which contains data of 170 AD patients with 257 interviews and 99 healthy controls with 243 interviews. The large bidirectional encoder representations from transformers (BERT_Large_) embedding with logistic regression classifier achieves classification accuracy of 88.08%, which improves the state-of-the-art by 2.48%.

**Conclusions:**

Using pre-trained language models can improve AD prediction. This not only solves the problem of lack of sufficiently large datasets, but also reduces the need for expert-defined features.

## Background

Alzheimer’s disease (AD) is the most common type of dementia which currently cannot be cured or reversed [1]. According to the World Alzheimer Report 2019, there were over 50 million people living with dementia in the world as estimated by Alzheimer’s Disease International (ADI), while the projected estimates for 2050 reach above 150 millions [2]. The common symptoms of AD include decreased awareness, disinterest in unfamiliar subjects, increased distraction, and speech problems [3]. However, if the disease is diagnosed in its early stage, a series of pharmacological and behavioral therapy approaches can be prescribed to reduce the pace or progression of the disease symptoms [4]. Clinical levels of cognitive impairment are categorized into 7 stages of: normal, normal ageing forgetfulness, mild cognitive impairment (MCI), mild AD, moderate AD, moderately severe AD, and severe AD [5]. In terms of observable linguistic symptoms, in the first three stages, the participants need more time to respond and find words, or have trouble to maintain focus on a conversation. In mild and moderate AD stages, patients have difficulty in understanding and explaining abstract concepts, completing sentences, and following long conversations. In the two most severe stages, patients cannot create grammatically correct sentences, almost lose the ability to understand words, and finally, become completely mute [5–7].

The healthcare industry has quickly realized the importance of data and as a result has started collecting them through a variety of methods such as electronic health records (EHR), sensors, and other sources. But analyzing these data and making decisions based on them is very time consuming and complicated. A large portion of this data is textual which makes the analysis more challenging. On the other hand, there is a large amount of information and hidden relationships in these textual data, and extracting this information is difficult for humans. In this regard, the use of machine learning and natural language processing (NLP) to analyze these data and inference based on the performed analysis has received increased attention. Moreover, according to the recent increasing power of deep learning techniques and their ability to extract complex relationships, employing these methods in medical text mining problems has been met with increased interest in recent years. Given the importance of the impact of AD on speech abilities of the patients, this study aims to develop a technique for AD risk assessment from transcripts of targeted speech elicited from the participants.

The task for acquiring speech data from the patients is the Cookie-Theft picture description test [8]. Initially, the test was used as a part of the Boston Diagnostic Aphasia Examination [8] assessment tool which was designed for diagnosing aphasia. Currently, the test is commonly used by speech-language pathologists to assess abnormal language production in patients with disorders such as aphasia, AD, right hemisphere lesions, schizophrenia, and etc [9]. In this test, an image is shown to the participant and they are asked to describe what they see in it. Generally, the Cookie-Theft image includes a mother washing the dishes in a sink while children try to steal cookies from a cookie jar.

Unlike most earlier studies, the features are extracted in our approach by the model itself in an unsupervised manner. As a result, more complex features are discovered and used for prediction. More precisely, the models are pre-trained on a large dataset to learn a good high dimensional (such as 1024 dimensions) vector representation for the input sentence or text, which will be used as input to AD versus healthy control (HC) classifiers. Another approach taken in this study to address the problem of insufficiently-sized datasets is text augmentation. Similar to most related works, the methods are evaluated on the Cookie-Theft picture description test transcripts of the Pitt corpus [10] from the DementiaBank [10] dataset. As mentioned earlier, the overall classification framework takes raw interview text as input. Our evaluation shows that pre-trained deep transformer-based language models with a simple logistic regression classifier work well in AD risk assessment and the results generally outperform those of the existing methods while the proposed method does not require any hand-crafted features for training the classifier.

### Related work

#### Feature-based approaches

For the first time, a computational approach to diagnosing Alzheimer’s disease using speech in English was introduced by Bucks et al. [11]. In that study, 8 AD and 16 HC participants were asked to speak about themselves and their experiences in 20–45 min sessions, and finally, some specific questions were also asked. Then, a number of linguistic features such as the noun rate, adjective rate, pronoun rate, and verb rate were extracted from the recorded speech and their distribution for the AD and control samples were used to train a classifier. Since then, many other studies have been conducted on this topic to improve the accuracy of AD prediction and study the various dimensions of AD (and other types of dementia) effects on speech. In general, most of these methods propose improvements based on increasing the number of expert-defined features [12, 13], increasing the number of participants [14], using acoustic features in addition to linguistic ones [15, 16], involving AD severity [14] and other types of dementia scores in classification [17], considering the impact of AD on other types of diseases [18], changing the interview’s structure [16], using linguistic impairment for predicting AD onset [19], and relating linguistic features to neuropsychological tests [19].

One of the most comprehensive studies on this topic was conducted by Fraser et al. [20]. In that study, an extensive categorization of linguistic features was presented, in which linguistic features were categorized into POS (part-of-speech) tags, syntactic complexity, grammatical constituents, psycho-linguistics, vocabulary richness, information content, repetitiveness, and acoustics. Also, the study categorized all different kinds of language disorders into the four groups of semantic impairment, acoustic abnormality, syntactic impairment, and information impairment. The paper collected 370 linguistic features from the data and reported the topmost 35 of these features for AD prediction.

In all earlier works, in order to automatically diagnose the disease using speech, information content units were introduced by human experts, and a classifier used them in order to predict the participant’s category. However, Yancheva et al. [21] and Sirts et al. [22] tried to enrich and enhance information content units of targeted speech by clustering pre-trained global vector (GloVe) [23] embedding of words used by AD and HC participants. Using the mentioned clusters, they introduced some cluster-based measures which were used along with a number of standard lexicosyntactic and acoustic features for AD prediction.

In languages other than English, Khodabakhsh et al. [24] and Weiner et al. [25] respectively examined the subject in Turkish and German. Also, Li et al. [26] and Fraser et al. [27] both focused on a multilingual approach for diagnosing AD using targeted speech. They respectively tried to improve the AD prediction in Chinese and French languages (in which the existing datasets were insufficient) using an English classifier trained on a larger English dataset.

#### Deep learning-based approaches

For the first time, Orimaye et al. [28] used a deep neural network to predict MCI using speech. Unlike most previous works, that study did not use any hand-crafted features and the raw transcripts were fed to the model. The dataset used in the study was part of the Pitt corpus of the DementiaBank dataset, comprising 19 MCI and 19 control transcripts of the Cookie-Theft picture description test. They trained a separate deep neural network language model for each category, and then calculated the likelihood of the text in both language models. Finally, the class of the model with higher probability was selected.

Karlekar et al. [29] also used a deep neural network model to diagnose AD using four types of interviews: the Cookie-Theft picture description, sentence construction, story recall, and vocabulary fluency which included an unbalanced 243 HC and 1017 AD transcripts. Three classifiers: a convolutional neural network (CNN), a long-short term memory recurrent neural network (LSTM-RNN), and a CNN-LSTM were trained, taking sentences as sequences of pre-trained word embedding. In addition to AD diagnosis, the authors interpret the models using activation clustering and first derivative saliency heat map techniques which cluster the most significant utterances. The research used a highly unbalanced dataset, rendering the results somewhat questionable as discussed in Sect. “Why not use the entire Pitt corpus?”.

Fritsch et al. [30] used two different auto-regressive LSTM-based neural network language models to classify AD and HC transcripts of the Pitt corpus from the DementiaBank dataset. After that, Pan et al. [31] worked on predicting AD using a stacked bidirectional LSTM and gated recurrent unit (GRU) layers equipped with a hierarchical attention mechanism. The overall model takes the GloVe word embedding sequence as input.

## Methods

In this study, the most challenging problem in developing data-driven (i.e., machine learning-based) methods for recognizing Alzheimer’s patients from speech transcripts is the lack of a large dataset. Currently, the largest available dataset is the Pitt corpus from the DementiaBank dataset, which contains 500 picture description interviews from the AD and control groups. For the mentioned reason, most of the earlier work was based on features designed by experts, as it was not possible to use models capable of learning informative features by themselves. In this study, we employ the idea of utilizing a highly pre-trained language model to address this issue. Moreover, data augmentation techniques are also utilized to alleviate the small dataset problem. Our implementation of these ideas is described next.

**Fig. 1:**
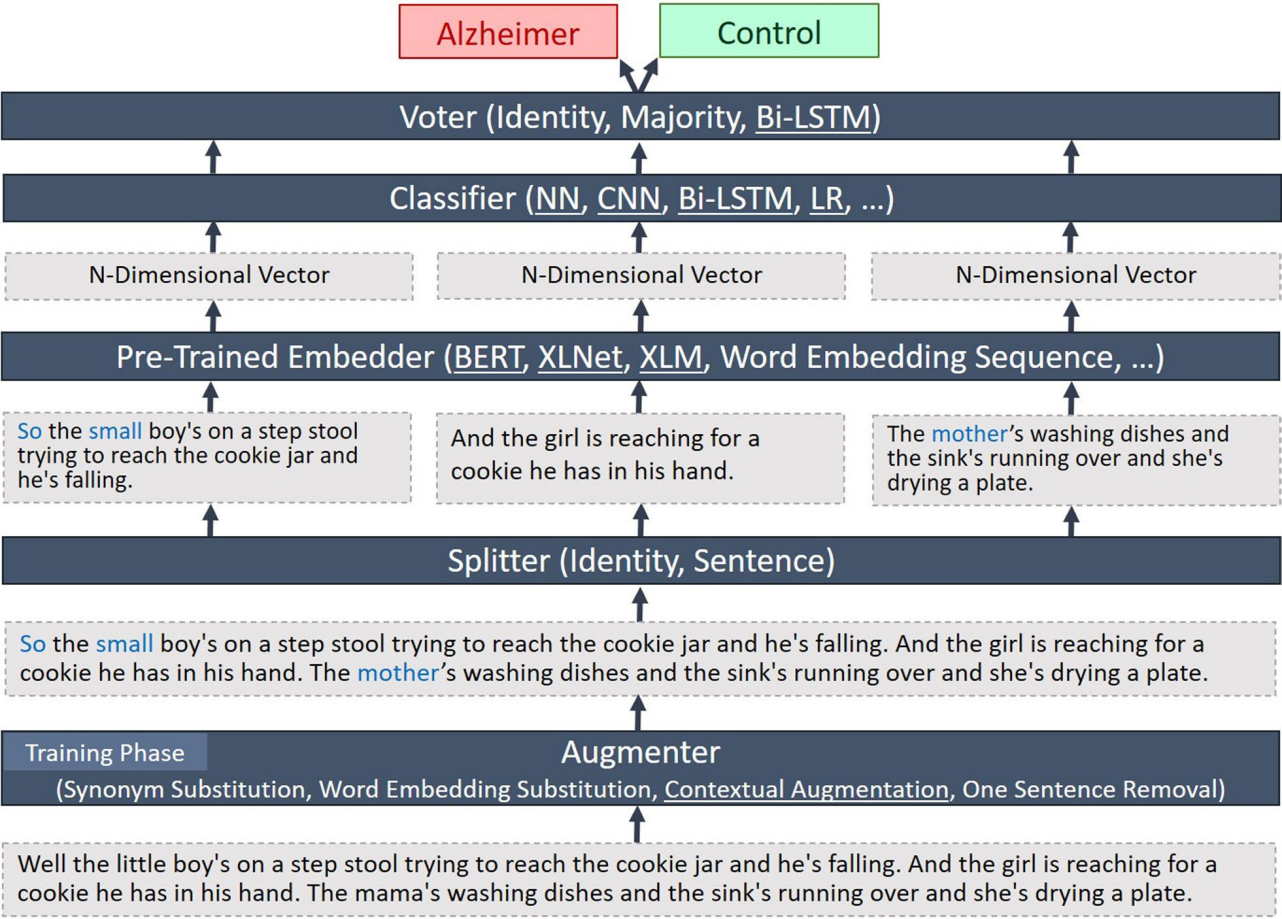
Overall classification procedure. The classification procedure consists of the steps of augmentation, splitting, embedding, classification, and voting, where augmentation is only used in the training phase. Also, when passing the entire transcript to the embedding layer, the splitting and voting layers are disabled. The underlined models are trainable here, and the others are fixed

**Fig. 2:**
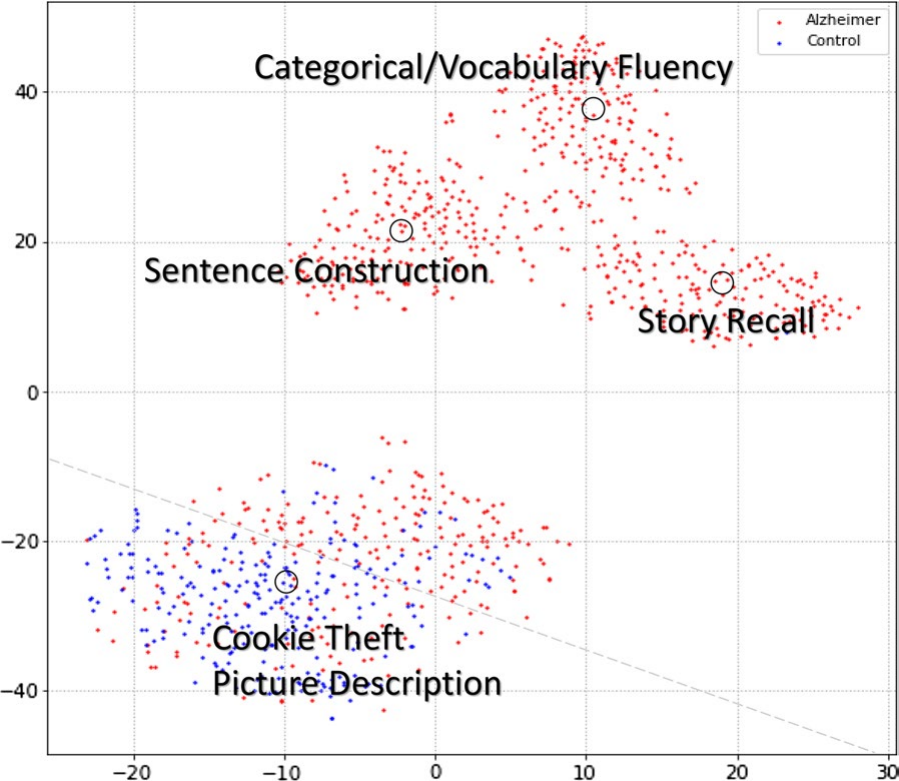
Visualized tSNE dimensionality reduction for the BERT_Base_ embedding of the entire Pitt corpus

**Fig. 3:**
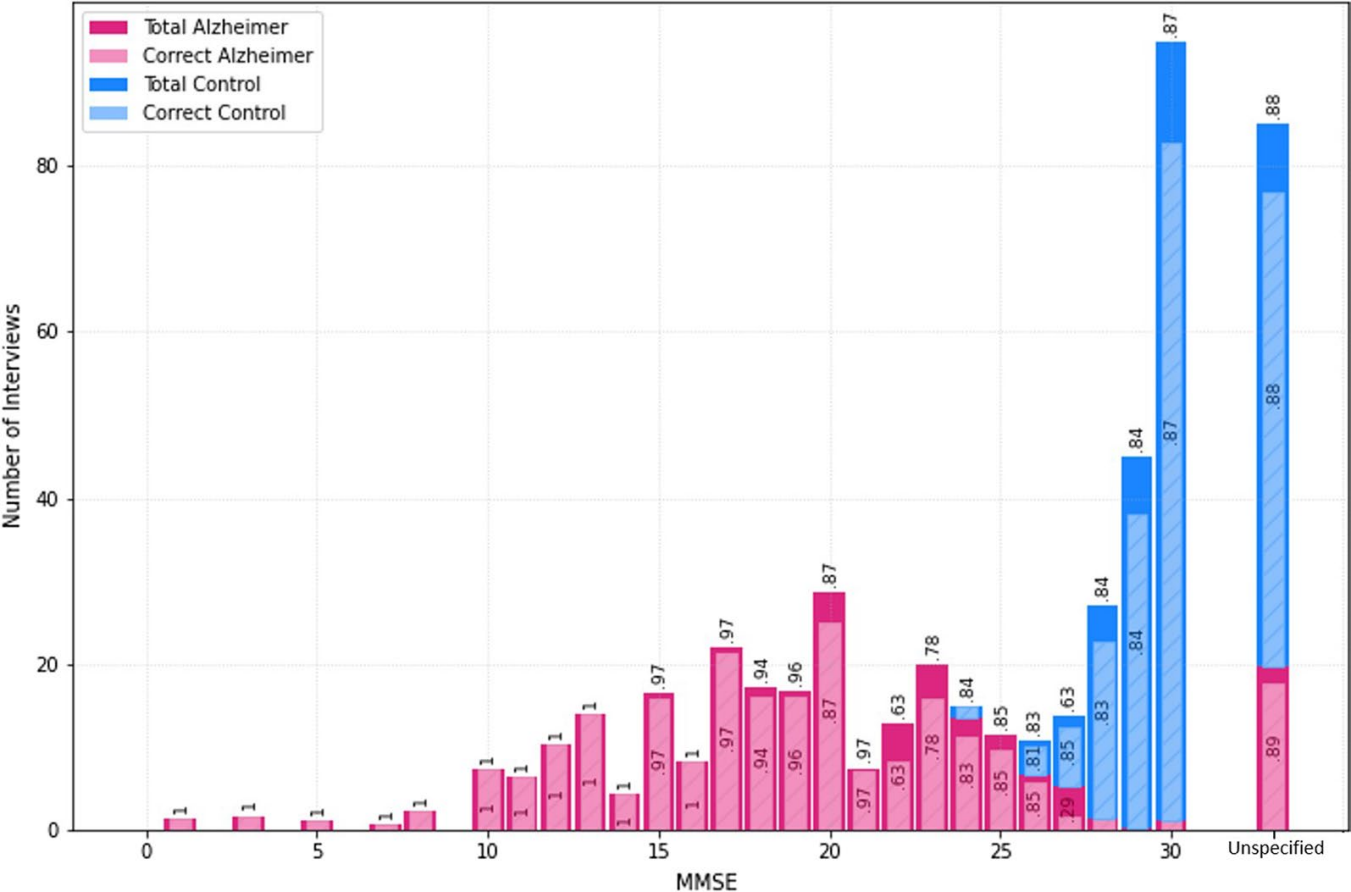
Mean 10-fold cross-validation classification accuracy, true positive rate, and true negative rate

### Overall classification framework

The overall process of classification is summarized in Fig. 1. The process consists of five layers. Each layer uses the output of the previous layer as input. The augmenter layer enriches the dataset with methods that will be introduced in Sect. “Dataset augmentation”. Note that this layer will be disabled in the test phase. The splitter layer is optional and chooses whether we want to process the whole text at once or break it down into sentences (and specify the final result by aggregating the results on sentences). It could be disabled by being set to the identity function when we intend to work on the whole transcript. The embedder layer embeds each input element (i.e. the entire transcript or a sentence) to a high-dimensional representation vector, and the classifier layer predicts the label of each embedded input. In fact, the classifier layer learns which of (and to what extent) the features that BERT (or other embedders) offers are suitable for predicting Alzheimer’s disease. Finally, if the classifier layer outputs multiple labels (that may happen when working on sentences), the voter makes the final decision using a majority voting mechanism. A layered architecture makes it much easier to combine different settings and understand the final model.

In our implementation, the augmenter and embedder layers are trained outside the classification framework and are only used there. Therefore, if there is a pre-trained embedding layer, training and inference will be done very quickly. Details on how to train these layers are explained in the following sections.

In this study, depending on the use of the splitter layer, two different approaches for classifying a transcript are implemented. In the first approach, the entire transcript is passed to an embedder and then the embedded transcript is directly classified. In this approach (from now on we will call it the text-level approach), the splitter and voter layers are disabled. In the second approach, the transcript is first split into sentences, and then these sentences are embedded and are subsequently classified. Finally, the label of the entire transcript is decided by majority voting on the labels of all sentences in the transcript. The second approach (from now on we will call it the sentence-level approach) is more compliant with pre-trained embedders since they are mostly pre-trained on single- or two-sentence inputs.

### Pre-trained deep language model

A model that defines a probability distribution over a sequence of words is called a language model. If a computational model wants to implement a language model, it is necessary to have a good understanding of the syntactic and semantic structures of that language. Therefore, using a model that has already learned a probabilistic distribution that correlates with these structures for classification reduces the need for large target-specific datasets. The transfer of knowledge from one model to another with a similar purpose is called transfer learning. We use transformer-based language models that have offered a breakthrough in many language understanding tasks in recent years [32]. The general flow of using a pre-trained language model for classification consists of three steps:

1. Unsupervised training of the general language model on a large dataset (such as Wikitext).
2. Unsupervised fine-tuning of the pre-trained language model on the target dataset (such as the Cookie-Theft picture description transcripts).
3. Using (with or without supervised fine-tuning) the target-specific pre-trained language model for the classification task.

To address the problems facing recurrent models such as the issue of short-term memory and the challenges facing the parallelization of training, Vaswani et al. [33] introduced transformers which consist of an extreme use of the attention mechanism that underpins many NLP models. The paper argues that the attention mechanism allows the model to focus on certain parts of the text for decision making. This functionality makes the attention mechanism useful for modeling biomarkers related to AD.

Al-Rfou et al. [34] used transformers for the first time as essential elements of a character-level language model. After that, Dai et al. [35] extended the model using relative positional encoding and segment-level recurrence. As a turning point in the transformer-based language models, we can refer to the bidirectional encoder representations from transformers (BERT) model proposed by Devlin et al. [36] at Google. In the training phase, the input sentence is masked, which means 15% of tokens are replaced with the [MASK] token, and the model tries to learn such representation or embedding for the context that considers both syntax and semantics to predict the masked token using the context. On the other hand, in the test phase the model takes in a raw sentence from one or multiple languages and returns a 768- or 1024-dimensional vector representation of the input text to be used as input to other classifiers such as LR, MLP, etc. An enhanced version of BERT for multilingual language understanding tasks was introduced by Conneau et al. [37], called cross-lingual language model (XLM), which benefits from using the translated language model (TLM) as well as the masked language model (MLM). Unlike BERT, XLM takes two related masked sentences from two different languages and tries to predict masked tokens using the same and the other language input sentences. This allows XLM to understand multilingual texts better. Also, BERT suffers from the train and test phase discrepancy and independent prediction of masked tokens. To correct this, Yang et al. [38] introduced an extended large network (XLNet) model based on a language model called Permutation Language Model.

The use of multilingual models offers a practical solution to the problem of lacking of a large dataset in many languages. As there is a limited collection of text data from Alzheimer’s patients in many languages, training a multilingual model in a source language (in which such large datasets are available) and applying it to making inference in the target language can offer a valuable solution. On the other hand, a number of language features that experts introduce are either specific to a particular language or their implementation may be different in different languages. Using multilingual models can also mitigate the need for such transfer of expert features between various languages.

In the current study, we use pre-trained BERT, XLNet, and XLM as deep networks for text embedding which convert raw participant transcripts/sentences to 768- or 1024-dimensional vectors. More precisely, to use these language models for the embedding layer described in Sect. “Overall classiffication framework﻿”, the entire transcript (in the text-level approach) or sentence (in the sentence-level approach) are passed to the model, and then, the last layer embedding of the [CLS] token is considered as the embedding of the entire input. The embedding models (which are used in this study as an embedder layer) are only passed through Phases 1 and 3 of the flow described earlier in this section. The reason for this is that the employed dataset is insufficient for unsupervised fine-tuning (of language models on the target dataset) even when using vast augmentation methods. In practice, using unsupervised fine-tuning is likely to have minimal impact on the overall performance of the model used in the current research (the effect of this feature on the results of the implemented model with the best performance is presented in Sect. “Evaluation results”). For the first phase, all embedding models are pre-trained with the corpus mentioned in the main article, and their implementation is taken from the HuggingFace transformers library [39].

### Dataset augmentation

Another approach to overcome the lack of access to large training input is dataset augmentation which means increasing the number of labeled samples of the dataset using some probabilistic or even heuristic algorithms. For example, the word *“beautiful”* in a sentence such as *“What a beautiful car!”* can be replaced with the word *“nice”* without changing the meaning of the sentence a lot. Augmentation in NLP can be done at different levels of linguistic units, and in this study, the word and sentence level augmentations are used for enriching the dataset. The most crucial challenge of augmentation in the text classification task is preserving the text class during augmentation. For example, a probabilistic model can replace *“beautiful”* with *“dirty”* in the mentioned sentence, which is grammatically and semantically correct but changes the sentence category. Two general approaches to augmentation have been used in this study, which are described below.

#### Similar word substitution augmentation

In this approach, a similarity measure must first be defined. The most obvious definition of similarity for words is the synonym relation which was first used in the field of deep learning by Zhang et al. [40] using the WordNet database [41]. Another common similarity measure is the inverse of the Euclidean distance or the Cosine similarity between word embeddings which was first used by Wang et al. [42]. In the mentioned methods, there is no guarantee of the correct grammar in the output sentence. It is also possible that the output sentence category changes by augmentation. For example, one of the markers of Alzheimer’s disease is the reduction in the vocabulary used in the conversation, so replacing a simple word like *“Delicious”* with its sophisticated synonym like *“Scrumptious”* can change the sentence category from patient to healthy and mislead the classifier. Another method that considers grammatical correctness along with the sentence context was introduced by Kobayashi [43] and is called contextual augmentation. In the contextual augmentation method, there is a language model which takes both the word’s context (i.e. the sentence that contains the word) and the whole sentence’s category and returns a probability distribution over all vocabulary. Augmentation is done by sampling from the returned probability distribution. Kobayashi [43] trained a Bi-Directional LSTM language model with this approach, and Wu et al. [44] enhanced the approach by using BERT as an underlying model.

All the mentioned methods were evaluated in this study, and the implementation was done using the NLPAug library [45] except for contextual augmentation for which the released code by the authors of [43] was used.

#### Sentence removal augmentation

Another ad-hoc approach which does not change the sentence category and also retains grammatical correctness is sentence removal. In this approach, one sentence is removed from the transcript, and it is expected that the output is still a valid transcript in the same category. Although it can be argued that the label may be changed by reducing the length of the text, considering the results of using or not using this idea, it is appropriate to use it in models that process the entire text at once (not sentence by sentence).

### Baseline models

In this study, in addition to the transformer-based models, bidirectional-LSTM and convolutional neural networks over the GloVe word embedding were also evaluated as baseline models to illustrate the advantages of pre-trained transformer-based deep language models over conventional deep models. In these models, the entire transcript is used as input. The reason for this decision is that unlike pre-trained models, there is no pre-training on single-sentence (or maximum two-sentence) texts and hence their training has to be done from the beginning. Therefore, splitting the transcript into sentences will not improve the performance of these models. In the CNN model, each transcript (truncated or padded to *T* number of words) is converted to a sequence of embedded words. Then the sequence is passed to a number of stacked convolutional and max-pooling layers followed by fully-connected layers and finally a sigmoid output layer that yields *P*(*AD*|*transcript*). Also, in the bidirectional-LSTM model, the embedded word sequence is passed to a number of stacked forward and backward LSTM cells followed by fully-connected layers and a sigmoid output layer in a similar fashion. Structurally, if we move forward in the CNN layers, the model tries to conclude more semantic features using spatially close features in the previous layer. But in the LSTM model that considers long range dependencies, an attempt is made to learn new compound features from features of all previous steps (or from features of the whole sequence in the bidirectional LSTM). The main weakness of this model is the forgetting of distant features (spatially) to produce new compound features. In both of these models, there is no attention mechanism.

### Experimental setup

In this section, we describe our implemented methods and their corresponding settings in the training and evaluation phases.

#### Implemented methods

For each layer of the overall framework, there were several options from which the following were implemented. For the augmenter layer, synonym-substitution and contextual augmentation were implemented along with ad-hoc sentence removal augmentation. As implemented by Kobayashi et al. [43], the corresponding language model used in contextual augmentation was a single layer bidirectional LSTM. For the splitter layer, in addition to the identity function, the sentence splitter was implemented for the sentence-level approach. For the pre-trained embedder layer, BERT (base and large), XLNet (base and large), XLM, and the GloVe word embedding sequence (50-dimensional version) were investigated. For the classifier layer, logistic regression, single hidden layer neural network, single-layer bidirectional LSTM, and three-layer CNN were examined. Finally, for the voter layer, in addition to the identity function, majority voting, and a single-layer bidirectional LSTM were implemented for the sentence-level approach. Although different combinations of layers were implemented, only significant cases of each group have been reported in Sect. “Evaluation results” .

#### Training settings

For the contextual augmentation, as implemented by Kobayashi et al. [43], the cross-entropy loss function and the Adam optimizer was used. The number of augmentations per transcript is a hyper-parameter for the augmentation layer. For the pre-trained embedding layer, only the HuggingFace transformers library [39] was used and no additional training was done on the implemented models. For the classification layer, binary cross-entropy was employed for the loss function and the Adam optimizer was used to minimize it. For the voter layer, only bidirectional LSTM was trainable for which, again the binary cross-entropy loss function and the Adam optimizer were utilized. All models were evaluated using 10-fold cross-validation without stratified sampling.

## Results

### Dataset

The models are evaluated on the transcripts of the Cookie-Theft picture description test of the Pitt corpus from the DementiaBank dataset, which contains 170 possible or probable AD patients with 257 interviews and 99 healthy control (HC) participants with 243 interviews.

**Table 1:** Demographics of Cookie-Theft picture description test of the Pitt corpus

	AD	HC
Participants	170	99
Samples	257	243
Age (years)	$71.7\pm 8.5$	$64.2\pm 7.9$
Gender (male/female)	87/170	88/155
Mini-mental state exam	$18.6\pm 5.1$	$29.1\pm 1.1$
Number of words	$100.9\pm 58.3$	$111.5\pm 57.2$

**Table 2:** The similarity between predicted health scores of the S-BERT_Large_-LR model and MMSE [49] scores

Measure		
Phase	Pearson correlation	Spearman’s rank correlation
Train	0.78	0.81
Validation	0.70	0.74

Most of the data were gathered as a part of the Alzheimer’s and related dementias study at the University of Pittsburgh School of Medicine between 1983 and 1988. The interviewer shows the participant the Cookie-Theft picture and asks him/her to state everything he/she sees in it. The audio records of all interviews were manually transcribed and annotated with POS-tags in the CHAT [46] format. Detailed demographics of the data is specified in Table 1.

### Why not use the entire Pitt corpus?

Some earlier studies based on the Pitt corpus (such as Kerlekar et al. [29]) used all the tests of the corpus including the Cookie-Theft picture description, story recall, sentence construction, and categorical/verbal fluency for classification purposes. The first problem with using the entire corpus is that the corpus is highly unbalanced (note that Table 1 only provides demographics of the Cookie-Theft picture description test from the Pitt corpus, which is perfectly balanced, although the whole dataset is unbalanced and the number of AD/HC samples are 846/244 in the overall corpus), and as a result, a naïve classifier that always outputs AD labels can achieve a classification accuracy of 78% on such a dataset.

The second problem is that except for the Cookie-Theft picture description test, the Pitt corpus was only administered to AD subjects for all the other tests, which means that the classifier might learn invalid features for AD prediction. For example, a classifier may just output an AD label by checking if the input is not from the Cookie-Theft picture description test, and otherwise, work as normal. Using this approach, a normal classifier with 80% accuracy can achieve approximately 92% accuracy on the whole Pitt corpus. Figure 2 provides an example of this problem. The figure shows visualized two-dimensional tSNE [47] diagram for the BERT_Base_ embedding of the entire transcripts of all tests in the Pitt corpus. According to the figure, the tests are completely differentiable, and as a result, the mentioned problem is quite probable to arise. Thus, in Sect. “Results”, studies based on the entire corpus were not included.

### Evaluation measures

The most well-known measure to evaluate classification is the accuracy score which is the fraction of predictions the model performed correctly. Most related studies have reported accuracy as the quality of their classification models and tried to improve this measure as an important goal. As discussed in the previous section, the accuracy measure alone does not provide a complete interpretation of the model performance (for example, high accuracy can be achieved using the entire Pitt corpus, while the model performance is not sufficient for practical use). Two other practical measures are precision and recall (also called sensitivity). In this study, precision is the number of correct AD predicted samples over the total number of AD predicted samples and recall is the number of correct AD predicted samples over the total number of AD samples. These two measures should be examined together and for this reason, the F_1_ score is defined. The F_1_ score is the harmonic mean of the precision and recall measures. A combined high precision and recall results in a high F_1_ score. In other words, highly imbalanced precision and recall indicates that the model has not an approximately equal performance for detecting all labels. All the aforementioned measures are in the range of zero to one, and can be reported as a percentage. Compared to the accuracy score, fewer previous studies have reported recall, precision, and F_1_ measures. In this study, all the introduced measures are reported to make it possible to compare our work more comprehensively with previous works.

### Compared methods

We compared the results of our models with all related studies that evaluated their models on the Cookie-Theft picture description test of the Pitt corpus. Therefore, the best models (according to the introduced performance measures) are selected for comparison. The first one is the method introduced in [20] which maintained the status of having the state-of-the-art accuracy score for several years. The second compared method was introduced by Yancheva et al. [21]. They tried to enrich and enhance human-supplied information content units by clustering GloVe embedding of frequent words of each category. After that, Sirts et al. [22] extended the idea of Yancheva et al. [21] by introducing propositional idea density features that work better on free-topic conversational speech. Hernández et al. [48] introduced 105 hand-crafted features and used them to train a support vector machine (SVM) classifier. They reported all the well-known and informative measures for the classification tasks and also achieved good results. Fritsch et al. [30] trained two different auto-regressive LSTM-based language models for each group and classified each transcript by calculating its perplexity on the models and selecting the model corresponding to the lowest perplexity. Currently, that study has the best recall and accuracy scores for AD versus HC classification on the target dataset. Pan et al. [31] utilized a stacked bidirectional LSTM and GRU recurrent units equipped with a hierarchical attention mechanism. Up to now, this study has the best precision and F_1_ scores for AD versus HC classification on the target dataset. The last two studies by Li et al. [26] and Fraser et al. [27] were focused on multilingual AD prediction and hence their main goal was not to improve the unilingual classification. Li et al. [26] used 185 lexicosyntactic features for a logistic regression classifier and Fraser et al. [27] utilized class-based language modeling and information-theoretic features for an SVM classifier.

**Table 3:** AD versus HC classification scores

Method	Embedding	Classifier	Precision	Recall	Accuracy	F_1_
Fraser et al. [20]	35 Hand-Crafted Features	LR	–	–	81.92	–
Yancheva et al. [21]	12 Cluster-Based Features + LS&A	Random forest	80.00	80.00	80.00	80.00
Sirts et al. [22]	Cluster+PID+SID Features	LR	74.4 ±1.5	72.5 ±1.2	-	72.7 ±1.2
Hernández et al. [48]	105 Hand-Crafted Features	SVM	81.00	81.00	79.00	81.00
Fritsch et al. [30]	One-Hot Word Embedding Sequence	–	–	**86**	85.6	–
Pan et al. [31]	GloVe Word Embedding Sequence	Bi-LSTM$\mid$GRU Hierarchical Attention	84.02	84.97	–	84.43
Li et al. [26]	185 Hand-Crafted Features	LR	–	–	77	–
Fraser et al. [27]	Info and LM Features	SVM	–	–	75	77
CNN + SSA	GloVe Word Embedding Sequence	CNN	76.38 ±8.49	77.47 ±8.97	76.48 ±5.88	76.36 ±5.91
BiLSTM + SSA	GloVe Word Embedding Sequence	Bi-LSTM	74.71 ±1.92	75.00 ±14.82	75.51 ±5.77	74.22 ±8.71
BiLSTM + CA	GloVe Word Embedding Sequence	Bi-LSTM	78.40 ±6.60	73.95 ±12.96	77.36 ±6.19	75.43 ±7.83
T-BERT_Base_-LR	BERT_Base_ (Text Level)	LR	85.09 ±3.11	78.69 ±8.35	82.76 ±3.74	81.51 ±4.73
T-BERT_Large_-LR	BERT_Large_ (Text Level)	LR	88.21 ±5.33	80.86 ±7.58	85.10 ±3.43	84.04 ±3.93
T-XLNet_Base_-LR	XLNet_Base_ (Text Level)	LR	84.74 ±6.31	79.26 ±7.72	81.92 ±5.88	81.75 ±6.19
T-XLNet_Large_-LR	XLNet_Large_ (Text Level)	LR	82.30 ±5.15	83.83 ±4.34	82.87 ±3.14	82.86 ±2.60
T-XLM-LR	XLM (Text Level)	LR	80.31 ±5.29	79.13 ±8.43	80.21 ±4.94	79.49 ±5.76
S-BERT_Base_-LR	BERT_Base_ (Sentence Level)	LR	90.31 ±7.36	76.52 ±8.06	84.46 ±6.31	82.72 ±7.21
S-BERT_Large_-LR	BERT_Large_ (Sentence Level)	LR	**90**.**57** ±3.18	84.34 ±7.58	**88**.**08** ±4.48	**87**.**23** ±5.20
S-BERT_Large_-LR-BiLSTM	BERT_Large_ (Sentence Level)	LR	89.06 ±5.19	77.71 ±7.33	85.19 ±4.92	83.61 ±5.69
S-BERT_Large_-BiLSTM	BERT_Large_ (Sentence Level)	BiLSTM	87.98 ±5.31	75.03 ±5.99	83.43 ±5.51	81.49 ±5.31
S-XLNet_Base_-LR	XLNet_Base_ (Sentence Level)	LR	83.19 ±6.39	74.34 ±8.12	80.00 ±5.48	78.32 ±6.16
S-XLNet_Large_-LR	XLNet_Large_ (Sentence Level)	LR	76.95 ±6.62	71.30 ±8.29	75.31 ±5.56	73.75 ±6.14
S-XLM-LR	XLM (sentence level)	LR	84.00 ±4.74	73.47 ±9.80	80.21 ±5.47	78.14 ±6.72

Other settings of the proposed framework with different classifiers or augmenters which did not have significant effects on the scores are not shown

**Table 4:** Two invalid predicted transcripts by the model with the best accuracy score (S-BERT_Large_-LR)

Transcript	Actual label	Predicted label	Predicted AD probability
And the boy in the cookie jar. And the girl reaching up to him. The stool slanting ready to topple. And the cookie jar is open. And the lid’s in there. And the door’s open. And mother’s drying the dishes and standing in a pool of water it looks water running down from the sink. ...	AD	HC	0.483
Okay. It was summertime and mother and the children were working in the kitchen. And the window was open and there was a slight breeze blowing in. Mother was daydreaming and forgot and left the water in the sink running and it was overflowing. The children were hungry and ...	HC	AD	0.532

Predicted AD probability ranges between 0 and 1

### Evaluation results

Table 3 reports precision, recall, accuracy, and F_1_ scores of the compared methods as well as those of the proposed methods in the framework introduced in this paper. The reported scores are averaged on a 10-fold cross-validation (without stratified sampling) procedure. Note that for the Fritsch et al. [30] method there is no such entity as a classifier and classification was performed by evaluating perplexity of input transcripts on the trained language models of both classes. As mentioned earlier, two different approaches have been implemented to use the pre-trained embedders, the first one is passing the entire text to the embedder (specified by a T- prefix in the method’s name) and the second one is passing each sentence of the text to the embedder separately (specified by an S- prefix in the method’s name). All the methods with the first approach have been enriched by the one-sentence-removal augmentation method. Furthermore, the CNN method is used with the synonym substitution augmentation (SSA) method and the BiLSTM is used with the SSA and contextual augmentation (CA) methods separately. The CA and SSA augmentations had almost no effect on the methods which used pre-trained language models, so they are not reported in Table 3. Also, for the model with the best accuracy score (S-BERT_Large_-LR), two additional versions with bidirectional LSTM classifier (S-BERT_Large_-BiLSTM) and bidirectional LSTM voter (S-BERT_Large_-LR-BiLSTM) are included.

As mentioned before, it seems that using unsupervised fine-tuning (using the MLM objective and next sentence prediction) on the Cookie-Theft picture description transcripts of the Pitt corpus does not have much effect on the results due to the lack of sufficient data for the target task. According to the experiments performed, using unsupervised fine-tuning for the model with the best accuracy score (S-BERT_Large_-LR in equivalent settings on average results in the accuracy and F_1_ scores of 87.89% and 86.11%, which are almost no different from the scores of a version without this feature (note that due to the fundamental differences of this approach with other models, we did not include it in Table 3).

Moreover, Fig. 3 illustrates the mean 10-fold cross-validation classification accuracy, true positive rate (the number of correct predicted AD samples over total number of AD samples, also called the sensitivity), and true negative rate (the number of correct predicted HC samples per total number of HC samples, also called the specificity) plotted versus the mini-mental state exam (MMSE) [49] scores of the participants. The figure helps us to see how the model works for detecting label of participants with different AD severity levels. The true positive rate for each MMSE score represents the model performance in detecting AD from actual AD patients in that score. Similarly, the true negative rate represents the model performance in detecting HC label from actual HC participants in that score. Totally, the accuracy score represents the model performance in detecting the correct label from both participant groups in the corresponding MMSE score. Numbers in the pink bars are true positive rates and in the blue bars are true negative rates. Also, the numbers on top of the bars are the total mean accuracy for that MMSE score. Note that all of the rates are scaled between 0 and 1. The MMSE scores were not reported in the dataset for some participants while their AD/HC labels were present. The results for these participants are grouped in the “Unspecified” bar in this figure.

In addition to classification, models such as logistic regression and neural networks with a sigmoidal final activation function can also output the AD probability (or 1—health probability) of the current input. Referring to the continuity of linguistic impairments from perfect health to severe AD, this probability can be interpreted as a correlated variable to the severity of the AD condition of the participant. Therefore, another approach for interpreting the models and evaluating them is calculating the similarity between their predicted health probability and the MMSE score, scaled between 0 and 1. The results using two common similarity measures, the Pearson correlation and Spearman’s rank correlation (which is the Pearson correlation on the samples’ ranking), are reported in Table 2. Both mentioned correlation measures are reported between − 1 and 1.

## Discussion

### Interpretation of results

According to Table 3, among the models that use only hand-crafted features, Fraser et al. [20] reports the best accuracy score, although it has not reported other evaluation measures. Among the baseline models introduced in our study (CNN + SSA, BiLSTM + SSA, and BiLSTM + CA), which are conventional deep neural network models, the contextual augmented version of bidirectional-LSTM achieved the highest accuracy score of 77.36%. However, even with the extreme use of augmentation methods these baseline models did not yield acceptable results compared to other methods. Overall, the sentence-level BERT_Large_ embedding of sentences passed to logistic regression (S-BERT_Large_-LR method) achieved the highest accuracy score (88.08%) among all the models introduced in this study as well as the models used in previous studies, and improved the accuracy score by 2.48% (equivalently 17.22% error-rate reduction). At the same time, this model achieved the best precision and F_1_ scores with 6.55% and 2.80% improvements, respectively. Still, Fritsch et al. [30] showed the best recall score with 1.66% difference although they did not report the F_1_ measure. The first advantage of our proposed methods compared to Fritsch et al. [30] is that we train a single language model for both the AD and HC groups which helps the model to use samples from both classes for the desired task. The other advantage is that our models are highly pre-trained on large datasets which enables them to start training on new, smaller datasets with good initialization parameters and also avoid overfitting.

Among the methods evaluated in this study, on average, the models based on the BERT family of embedders worked better than the others. Although XLNet has historically been designed to address BERT problems, BERT and its derivatives still perform better in many activities [32]. Moreover, employing word-level augmentation techniques along with pre-trained deep language models did not improve results (and hence the evaluation of their versions with augmentation was not reported in Table 3).

Table 2 shows that the best model has a Pearson correlation of 0.78 and 0.70 for the train and validation phases, and a Spearman’s rank correlation of 0.81 and 0.74 for these phases between the health score and the MMSE score, indicating that the model has learned useful patterns for classification. Based on the reported similarity measures, it can be concluded that on average the MMSE score and our model’s health score are linearly correlated. This is indeed an advantage for the proposed model in that while the MMSE score [49] is obtained through a detailed interactive exam that evaluates visuospatial, executive, naming, memory, attention, language, abstraction, delayed recall, and orientation cognitive skills, the data collection task involved in the Cookie-Theft picture description test used in our model is a simple and short pseudo-conversational procedure. Interestingly, the results obtained in this section are related to the reported results in the work of Eyigoz et al. [19]. The objective of that study was to use linguistic markers to predict the onset of Alzheimer’s disease in cognitively normal individuals. The study’s experiments showed that the stated goal is possible to achieve and, in fact, using models based on linguistic variables performed better than a predictive model based on non-linguistic variables (such as neuropsychological test results, age, gender, APOE $\varepsilon 4$ alleles, etc.). The results of this section show that the severity of linguistic impairments is highly correlated with the estimates based on non-linguistic variables (corroborating the results reported by Eyigoz et al. [19]).

In this study, neural network interpretation methods were not used but in Table 4, two false negative and false positive classification errors are reported. In comparison, it is almost clear that the first sample has less grammatical fluency but both samples refer to similar information elements. In the S-BERT_Large_-LR model, the predicted AD probability is the mean of logistic regression classifier outputs for each sentence of the transcript. The important point is that in both samples, the predicted AD probabilities are very close to 0.5 which can be interpreted as that the model has not learned a wrong feature, rather, it has not learned a proper feature to predict AD from the reported samples.

### Advantages and limitations

As mentioned in Sect. “Pre-trained deep language model”, the proposed approach takes advantage of the powerful pre-trained language models that attempt to learn the structure and features of the language from a large dataset, and only uses the target dataset to learn how to use these features for AD prediction. This not only reduces the need for expert-defined language features, but also makes it possible for more complex features to be extracted from the data. The next advantage of sentence embedding models is that they consider the entire raw text and there is no out-of-context word embedding layer that would convert each word to a representation vector without considering its context.

As mentioned earlier, even using augmentation methods, the largest currently available dataset for AD prediction is still insufficient in size for unsupervised fine-tuning (Second phase specified in Sect. “Pre-trained deep language model”) large transformer-based language models (e.g., BERT_Large_ has 340 million parameters). But if there is a large enough dataset, using language model fine-tuning, our approach can extract more complex and context-related features while the models based on expert-defined features can only choose from a limited set of predefined features.

The most important limitation of the current study that needs to be addressed in the future is that it is difficult to use common neural network interpretation methods due to the large number of model parameters. Using interpretation, we can understand why the model predicts a wrong label for a transcript. Also, in the case of a correct prediction, we can identify language features that the network has paid more attention to. This is particularly useful for studying Alzheimer’s disease as such interpretation can reveal important attributes of the speech which can most effectively discriminate between the participant groups.

Although using deep embedding models instead of expert (linguistic) features can improve the performance by extracting more complex relationships, it does not provide clear features tied to clinical practice that can be validated easily as opposed to expert features. In this regard, a suggested solution is to use interpretation techniques. But the training phase must also be conducted in such a way that the extracted features are both interpretable and relatively sparse so that they could be validated clinically.

### Future work

One of the most popular types of transformer-based language models is the class of multilingual models. With a proper use of multilingual models, similar to approaches by Li et al. [26] and Fraser et al. [27], the problem of lacking access to a large dataset in one language can be addressed by transferring the knowledge of AD prediction from another language in which a large dataset is available. Using such transfer, the need to define linguistic features by experts in the target language is also addressed. In future work, we aim to improve multilingual AD prediction using pre-trained multilingual transformer-based language models along with cross-lingual transfer learning.

## Conclusions

According to the results of earlier studies, Alzheimer’s disease affects speech in the form of syntactic, semantic, information, and acoustic impairments. We employed a transfer-learning approach to improve automatic AD prediction using a relatively small targeted speech dataset without using expert-defined linguistic features. We evaluated recently developed pre-trained transformer-based language models that we enriched with augmentation methods on the Cookie-Theft picture description test of the Pitt corpus. Using sentence level BERT_Large_ with a simple logistic regression classifier, the accuracy and F_1_ scores of 88.08% and 87.23% were achieved which improved the state-of-the-art results by 2.28% and 2.80%, respectively. Pre-trained language models are available in many languages. Hence, the approach in this paper can be examined in languages other than English as well. Also, with the multilingual versions of these models, the knowledge of AD prediction in one language can be transferred to another language in which a sufficiently large dataset does not exist.

## Data Availability

The data (Pitt corpus from the DementiaBank dataset) that support the findings of this study are available from the TalkBank project by request from the dataset publisher.

## Abbreviations

AD: Alzheimer’s disease
ADI: Alzheimer’s disease international
BERT: Bidirectional encoder representations from transformers
CA: Contextual augmentation
CNN: Convolutional neural network
XLM: Cross-lingual language model
XLNet: Extended large network
GRU: Gated recurrent unit
GloVe: Global vector
HC: Healthy control
LSTM: Long-short term memory
MLM: Masked language model
MCI: Mild cognitive impairment
MMSE: Mini-mental state exam
NLP: Natural language processing
POS: Part-of-speech
RNN: Recurrent neural network
TLM: Translated language model
SVM: Support vector machine
SSA: Synonym substitution augmentation
